# Evaluation of Tracheal Stenosis in Rabbits Using Multispectral Optoacoustic Tomography

**DOI:** 10.3389/fbioe.2022.860305

**Published:** 2022-03-04

**Authors:** Yanting Wen, Dan Wu, Jing Zhang, Shixie Jiang, Chunyan Xiong, Dan Guo, Zihui Chi, Yi Chen, Lun Li, Ying Yang, Ting Liu, Huabei Jiang

**Affiliations:** ^1^ School of Computer Science and Technology, Chongqing University of Posts and Telecommunications, Chongqing, China; ^2^ Department of Ultrasound Imaging, The Fifth People’s Hospital of Chengdu, Chengdu, China; ^3^ Department of Psychiatry and Behavioral Neurosciences, Morsani College of Medicine, University of South Florida, Tampa, FL, United States; ^4^ Department of Medical Engineering, University of South Florida, Tampa, FL, United States

**Keywords:** trachea, vivo, multispectral, imaging, optoacoustic tomography

## Abstract

**Objective:** Photoacoustic tomography (PAT) and multispectral optoacoustic tomography (MSOT) are evolving technologies that are capable of delivering real-time, high-resolution images of tissues. The purpose of this study was to evaluate the feasibility of using PAT and MSOT for detecting histology in a rabbit tracheal stenosis model.

**Method:** A total of 12 rabbits (9 stenosis and three control) were randomly divided into four groups (A, B, C and D). Each group consisted of three rabbits, which were staged at the first, fourth, and eighth weeks of stenosis progression, respectively. PAT/MSOT images and corresponding histology from these experimental animals were compared, for analyzing the morphologic features and quantitative tracheal measurements in different tracheal stenosis stage.

**Result:** Both the PAT images and corresponding histology indicated the most severe degree of stenosis in group C. MSOT images indicated notable differences in tracheal contents of group B and D.

**Conclusion:** This study suggests that PAT/MSOT are potentially valuable non-invasive modality which are capable of evaluating tracheal structure and function *in vivo*.

## Introduction

Photoacoustic tomography (PAT), is an emerging biomedical imaging modality that combines optical and ultrasonic imaging for the combined benefits of enhanced optical resolution and acoustic penetration depth (Attia et al., 2019; Manohar and Gambhir, 2020). Multispectral optoacoustic tomography (MSOT) is based on the principle of PAT, which is formation of sound waves following light absorption by multi-wavelength pulsed laser. Due to different tissue contents respond to laser light energy differently, MSOT distinguishes absorbers based on their spectral signatures, allowing differentiation of either endogenous material (hemoglobin, melanin, lipids, and water) or exogenous contrast agents (Ntziachristos and Razansky, 2010; Li et al., 2018).

In recent years, tremendous developments in PAT have been achieved, allowing a wide range of applications in preclinical and clinical research, including imaging of thyroid (Dima and Ntziachristos, 2016; Roll et al., 2019), breast (Xu et al., 2019; Yang et al., 2020), tumors (Li et al., 2015; Yamada et al., 2020; Karmacharya et al., 2021; Knorring and Mogensen, 2021; Wang et al., 2021) and skin lesions (Petri et al., 2016; Dahlstrand et al., 2020), as well as functional neuroimaging (Wang et al., 2003; Wu et al., 2019a; Lv et al., 2020). More recently, MSOT is found to be a method with a multitude of possible applications ranging from the small animal studies, to clinical imaging applications (Taruttis et al., 2013; Anne et al., 2020; Karlas et al., 2021), indicating its ability of providing potent structural, functional, and molecular information. Furthermore, PAT and MSOT could be an accurate imaging modality for assessment of blood vessels diseases *in vivo* (Yang et al., 2019; Bok et al., 2021; Karlas et al., 2021), highlighting the potential of them as a new tool to evaluate structural and functional information in tubular structures like artery, vein, trachea and bowel.

Airway structure and function are key aspects of respiratory physiology across all ages. The accurate analysis of dimensions is crucial to assess tracheal physiological and pathological states, like chronic airway remodeling, tracheal contracture and stenosis (Ahmad and Yahaya, 2012; Both et al., 2021; Kornas et al., 2021). Recently, the number of cases of tracheal stenosis has therefore been increasing. Among 63 cases of benign central tracheal stenosis reported by Kleiss (Kleiss et al., 2013), 52 cases (83%) occurred after tracheotomy and tracheal intubation. Additionally, the fatality of immature infants has been mounting, resulting in an increased number of children that require prolonged intubation in neonatal ICUs. Especially in the threat of COVID-19, prolonged intubation causes tracheal wall damage in COVID-19 pediatric patients reported by Alturk (Alturk et al., 2021), which led to tracheal stenosis. Stenosis length, severity, and location are essential in the management plan. Although optical endoscopy is the standard diagnostic method for tracheal stenosis, it is invasive, with a high risk of causing severe infection.

The establishment of experimental animal models of granulation tissue-induced tracheal stenosis can simulate the entire pathophysiological process of tracheal stenosis caused by tracheal incision, which can closely mirror what occurs in clinical practice. Rabbits are most suitable for stenosis model based on previous studies (Mahmood et al., 2006; Nakagishi et al., 2008), given the length of the trachea of rabbits is similar to that of humans (70–85 mm) and has no obvious correlation with body weight. Moreover, its cartilage rings are similar to humans, with approximately 48–50 C-shaped cartilage rings.

Currently, there are several methods for evaluating airway stenosis, including invasive methods such as bronchoscopy or optical coherence tomography (OCT), and non-invasive methods such as ultrasound, computed tomography (CT), and magnetic resonance imaging (MRI). Due to the gas scattering characteristics of ultrasound, it is difficult to display the overall structure of the trachea (Gómez-Tamayo et al., 2018). Although CT (Cao et al., 2020) and MRI (Elders et al., 2021) are routine techniques for detection of tracheal stenosis, these methods confer a risk of ionizing radiation (especially for neonates) or are commonly unable to be utilized due to the presence of stents or metallic foreign bodies. Endobronchial OCT, as a new method for airway detection, has previously demonstrated the ability of delineating airway subcomponents in both animals (Mahmood et al., 2006; Lin et al., 2013; Lee et al., 2014) and humans (Balakrishnan et al., 2018; Goorsenberg et al., 2018; Kozlowski et al., 2019). However, as OCT is applied through an endobronchial tube, it may cause airway mucosa injuries or aggravate airway stenosis.

The purpose of our current study was to investigate the potential utility of PAT/MSOT in structurally and functionally detecting tracheal stenosis in real-time. To this end, an animal model of benign tracheal stenosis was imaged by PAT/MSOT during the first 1–8 weeks’ in stenosis development, involving structural, histological and functional changes of trachea and surrounding tissues. This study, to the best of our knowledge, represents the first noninvasive assessment of trachea physiology and pathology using PAT/MSOT.

## Materials and Methods

### Experimental Set-Up

**FIGURE 1:**
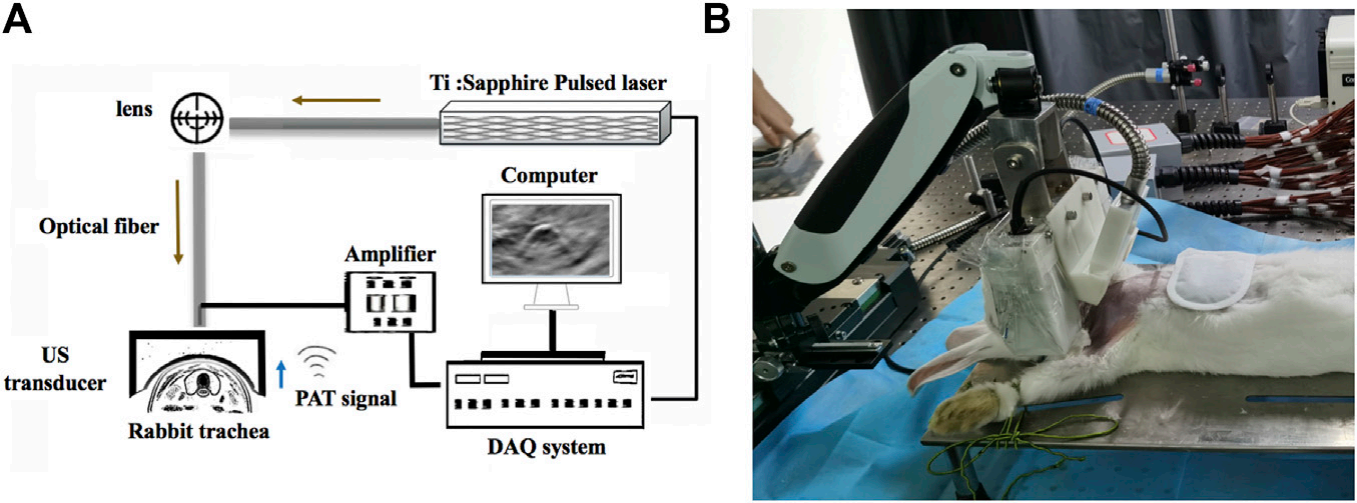
Schematic of the PAT/MSOT system for rabbit airway imaging. **(A)** The schematic of the hardware components employed, including the pulsed laser, optical fiber, ultrasound (US) transducer, computer, data acquisition (DAQ) system, and amplifier. **(B)** photograph of our system being utilized in a rabbit model.

We devised a PAT/MSOT system (Wu et al., 2019b) to enable noninvasive, real-time visualization of rabbit trachea as schematically shown in Figure 1. In this system, laser illumination was provided by an OPO Nd:YAG pumped optical parametric oscillator (Surelite, Continuum, CA, United States; wavelength range: 680–950nm; frequency 20 Hz). Light was transmitted to the imaging object through an optic fiber bundle. PAT signals were received by a 128-element concave transducer array (5 MHz central frequency) and transferred to a 64-channel data acquisition system (model 5,105, 12bit sampling progress, sampling frequency 50 MHz) with signals multiplexed in the acquisition progress (Figure 1A). This concave-shaped transducer array were arranged in a half arc (180° spanning range) with a radius of 50 mm here. The demonstrated spatial resolution was ∼150 μm with the time resolution of 10 ms. Depending on the transducer configuration and its concave array, the image depth of view was demonstrated to be 25 mm at least. In order to avoid physiological movements of the trachea (e.g., movement caused by circulation) and to improve the signal-to-noise ratio, ten times the signal averaging was applied (as verified in previous studies (Wang, 2009)).

The rabbits were placed on an animal holder allowing the whole trachea to be covered by the optic fiber. The focused transducer was set exactly at the same plane to obtain cross-sectional configurations at all points for ultrasound detection (Figure 1B). The entire scanning time was 15s and the laser energy density delivered to the animal was ∼22 mJ/cm^2^ (notably far below the American National Standards Institute safety limit of 100 mJ/cm^2^ in the near-infrared region (Wang, 2009)). PAT and MSOT images of the trachea were then generated using a LabVIEW program.

### Animal Models

The study protocol and animal recruitment were approved by the Ethics Committee for Laboratory Animals of the Chongqing University of Posts and Telecommunications, Chongqing, China. Twelve male New Zealand white rabbits (9 stenosis and three control), 4 months old and weight 2.5–3.5 kg were utilized for our study. The rabbits fasted 4 h before the test and were anesthetized with subcutaneous injections of 5% chloral hydrate (3–4 ml/kg). Once anesthetized, the rabbits were placed and fixed in a supine position. Rabbit models of airway stenosis were prepared as according to previously reported methods (Nakagishi et al., 2005; Zhang et al., 2020). After making a midline skin incision in the anterior neck, the larynx and trachea were exposed. The incision point was located 2–3 cm caudal to the inferior edge of the cricoid cartilage. A nylon brush (out-diameter, 7 mm) was inserted at the incision point into the trachea in the direction toward the mouth and removed after ten repetitions of scraping the tracheal wall. In order to avoid residual sutures affecting the imaging in the detection area, the incision was sutured layer by layer with sterile absorbable sutures (Size 5/0, average absorbable period: 5–7 days). We separated the rabbits into four groups for our experiment. Each group consisted of three rabbits, which were staged at the first, fourth, and eighth weeks of stenosis progression, respectively. Group A consisted of our control group, while B-D contained our rabbits with airway stenosis.

### PAT and MSOT Images Processing

The rabbits were imaged at different axial locations along the trachea, using our PAT/MSOT system as described above (Wu et al., 2019a). MSOT images were acquired at three wavelengths: 760 nm, 840 nm and 910 nm. After the experiments completed, the original PAT/MSOT data were post-processed by a MATLAB software. Tracheal data including wall thickness (TWT), cross-sectional area (CSA) and stenosis rate (SR) were measured by two members of our research team (WY and ZJ), respectively. Additionally, a maximum amplitude projection image was obtained by a multispectral reconstruction algorithm proposed by Huabei Jiang (Jiang, 2015). This algorithm was used to analyze the deoxygenated (HbR), oxygenated hemoglobin (HbO_2_) and HbT (Prahl, 2005), along with collagen (Col) content.

### Histological Examination

At the completion of our experiments, the rabbits were executed using an overdose of intravenous 10% chloral hydrate, and their tracheas were removed. The TWT, CSA and SR of these tracheal tissues were measured at 2–3 cm caudal to the inferior edge of the cricoid cartilage in the control group and at the incision area in the stenosis group. These specimens were subsequently maintained in an isotonic saline solution and placed in formalin for a period of 48 h, followed by standard paraffin embedding. Then the tissues were cut into 6-μm sections, and assessed by Hematoxylin and Eosin (H&E) and Masson staining. Images of these histologic specimens were reviewed under a microscope with ×40/×100 magnifications.

### Statistical Analysis

Data were expressed as means and standard deviations. Data analysis and mapping were analyzed by an R language system (Tidyverse package). PAT measurement inter-observer reproducibility was calculated using the correlation scatter plots by the ggplot two package. A paired two-tailed *t*-test and base function were performed for statistical comparison among different groups. *p* values of less than 0.05 were considered statistically significant.

## Results

### Modeling Success Rate

Ten rabbits underwent stenosis modeling. One rabbit in group D was excluded from the study due to inability to obtain reliable PAT images. This rabbit had developed white viscous pus scattered between the soft tissue layer of the anterior neck region and the tracheal incision, causing edematous tissue and displacement of the trachea. The modeling success rate was 100%, 100% and 75% in group B, group C, and group D, respectively.

### Rabbit Airway Detection by PAT/MSOT

#### PAT Imaging of Airway

**FIGURE 2:**
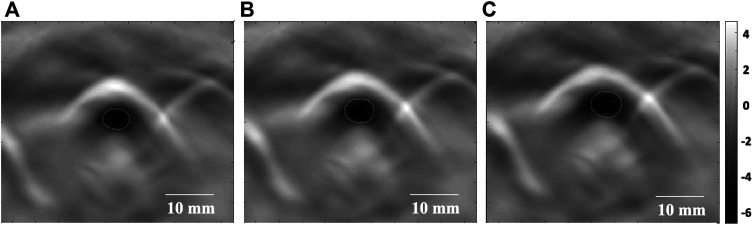
Real-time PAT imaging of the trachea at different phases of a respiratory cycle. **(A)** The smallest tracheal CSA (white circle) at the end of exhalation. **(B)** The tracheal CSA (white circle) begins to increase as inhalation begins. **(C)** The tracheal CSA (white circle) increases further at the end of inhalation. CSA: cross-sectional area. Dotted line: tracheal lumen. Scale bars are 10 mm long.

**FIGURE 3:**
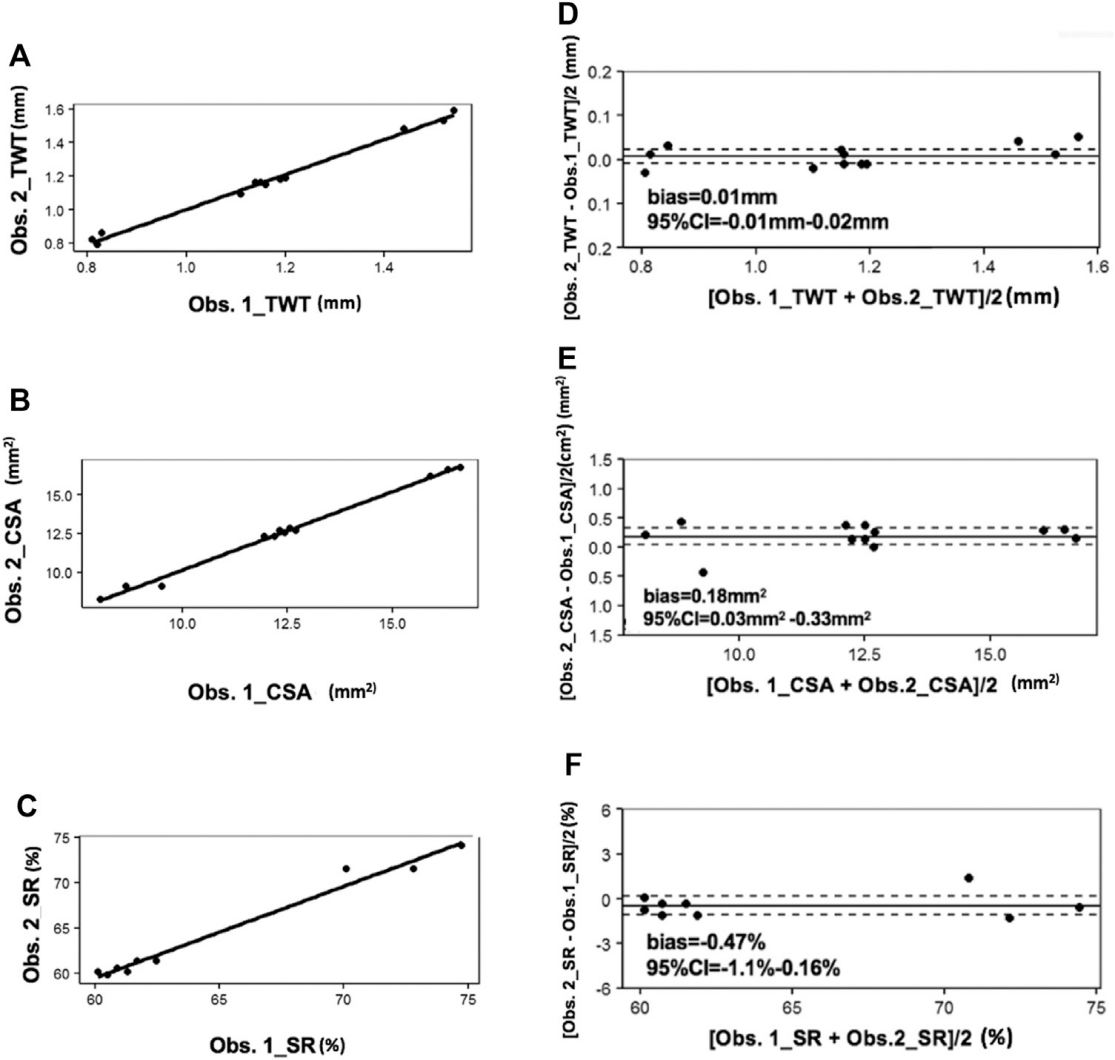
Correlation scatter plots for PAT between two observers. In **(A–C)**, solid bold lines represent the linear fitting curve. In **(D–F)**, the solid lines represent bias and dotted lines represent the 95%CI. CI: confidence intervals; Obs: observer.

**FIGURE 4:**
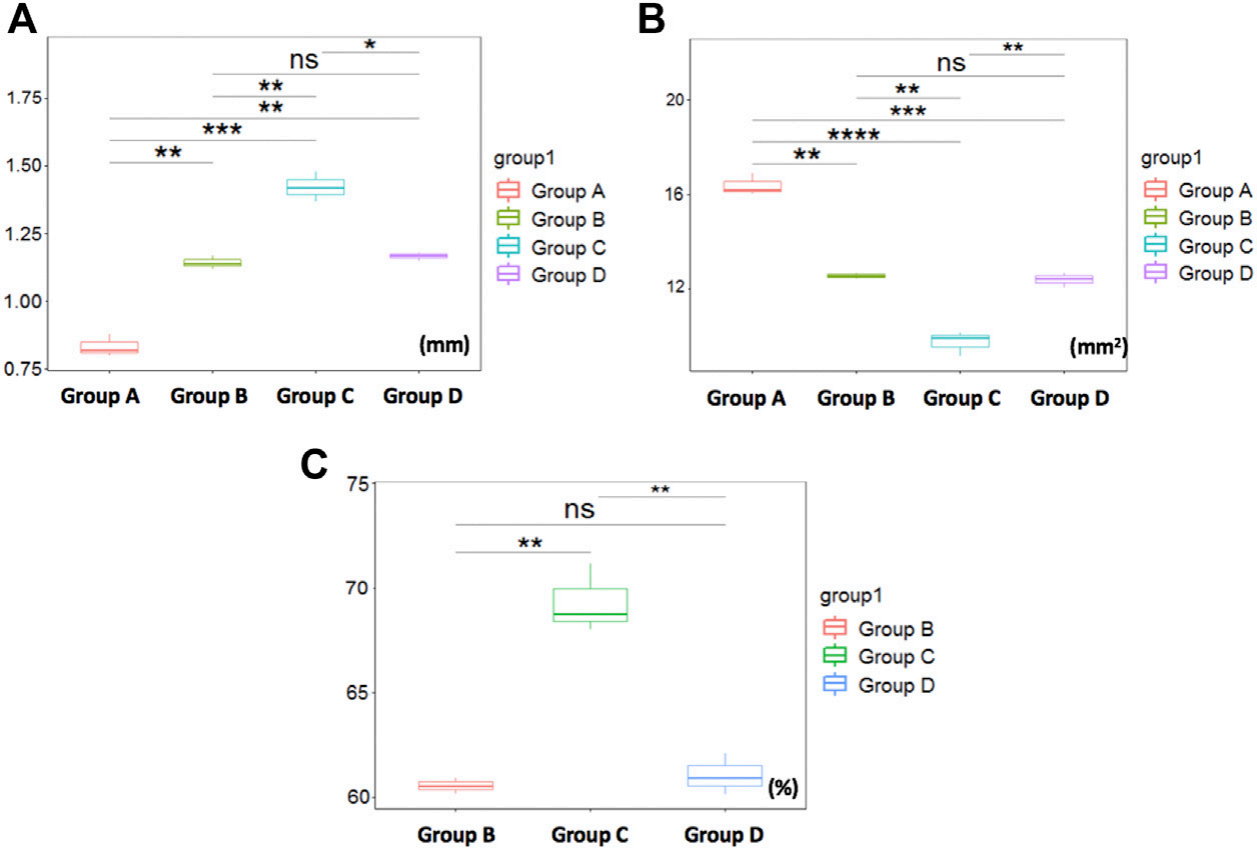
Comparison of tracheal measurements by PAT. **(A)** The TWT values in group C can be easily visualized as larger than in groups A^***^, B^**^, and D ^*^, respectively. **(B)** showing CSA in group C was decreased most among the four groups, compared with group A^****^, B^**^ and D^**^, respectively. **(C)** showing SR in group C was most severe among the stenosis groups, compared with group B^**^ and D^**^. Otherwise, no significant differences were found between group B and D measurements. ^*^ *p* < 0.05, ^**^ *p* < 0.01, ^***^ *p* < 0.001, ^****^ *p* < 0.0001, ^ns^p>0.05.

Images of high quality, with minimal artifact, were reliably obtained from all the animals. Therefore, all datasets were included for the final analysis. Rabbit breathing rhythms and their synchronous tracheal PAT images were recorded as follows: 1) Real-time PAT images of the normal control group (Group A) showed clear periodic variation of CSA in a respiratory cycle (Figure 2), while CSA variation in the stenosis groups were not apparently visualized from real-time PAT images; 2) At the end of exhalation, the smallest tracheal CSA of a respiratory cycle was observed (Figure 2A); 3) As the inhalation beginning, the tracheal CSA became to increase gradually (Figure 2B); 4) At the end of inhalation, the tracheal CSA increased further (Figure 2C).

#### PAT Measurements of Trachea

##### Inter-observer Reproducibility of PAT Measurements

The acquired PAT images were subsequently analyzed to determine the tracheal measurements by two members of our research team (WY and ZJ), including TWT, CSA and SR (Supplementary Table S1). In Figure 3A–C, solid bold lines represent the linear fitting curve. There were strong and significant correlations between the two observers for TWT (r = 0.96), CSA (r = 0.96) and SR (r = 0.88) quantified by correlation scatter plots. In Figure 3D–F, the solid lines represent bias and dotted lines represent the 95% confidence intervals (CI). For TWT measurement by two observers, Bias = 0.01 mm, 95%CI = -0.01 mm–0.02 mm; For CSA measurement by two observers, Bias = 0.18 mm^2^, 95%CI = 0.03 mm^2^ ∼ 0.33 mm^2^; and for SR measurement by two observers, Bias = -0.47%, 95%CI = -1.1%–0.16%.

##### Comparison of Tracheal Measurements by PAT

The intergroup comparison was applied by the average of the two observer measurements (Figure 4). The mean (SD) TWT above the tracheal bifurcation in the control group (group A) was 0.82 ± 0.02 mm, whereas that of the stenosis groups (group B ∼ D) were 1.14 ± 0.04mm, 1.50 ± 0.30mm, 1.17 ± 0.11mm, respectively. In addition, the TWT of stenosis groups were significantly larger than the control group (*p* < 0.05). The mean (SD) CSA above the tracheal bifurcation in the control group (group A) was 16.28 ± 0.33 cm^2^, whereas that of the stenosis groups (group B ∼ D) were 12.37 ± 0.30 cm^2^, 8.78 ± 0.92 cm^2^, 12.47 ± 0.27 cm^2^, respectively. The CSA of stenosis groups were significantly decreased compared to the control group (*p* < 0.05). The mean (SD) SR above the tracheal bifurcation in the stenosis groups (group B ∼ D) were 60.88 ± 0.74mm, 72.47 ± 1.84mm, 60.77 ± 0.73mm, respectively. Among all the measurements of the three stenosis groups, statistically significant differences were found between groups B and C (*p* < 0.05), as well as between groups D and C (*p* < 0.05), while no statistically significant differences were found between measurements comparing group B and D (*p* > 0.05). The TWT values in group C can be easily visualized as larger than in groups A, B, and D (*p* < 0.001, 0.01 and 0.05, respectively) (Figure 4A). Among all the measurements values recorded by PAT, the CSA in group C was decreased most among the four groups, compared with group A, B and D (*p* < 0.0001, 0.01 and 0.01, respectively) (Figure 4B). SR in group C was most severe among the stenosis groups, compared with group B (*p* < 0.01) and D (*p* < 0.01), respectively (Figure 4C).

#### PAT and Histology

**FIGURE 5:**
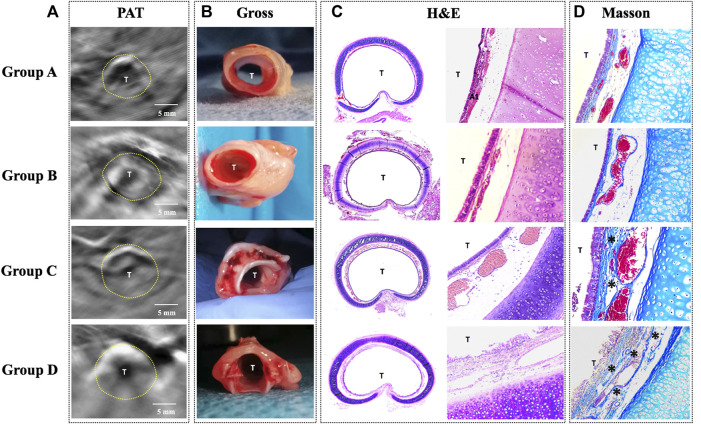
Comparison of PAT images and the corresponding histology in four groups. **(A)** PAT image acquired at 760 nm wavelength. **(B)** Gross tissue specimen. **(C)** Histology of the cross-sectional trachea with HE staining (×40/×100). **(D)** Histology of the trachea with Masson staining (×100) showing increased collagen distribution (*). PAT, photoacoustic tomography; Gross, gross examination; H&E, hematoxylin and eosin staining; Masson, Masson staining; T, tracheal lumen Dotted line: trachea. Scale bars are 5 mm long.

Histology in all rabbits via H&E and Masson stain revealed the following (Figure 5): 1) Normal tracheal in group A; 2) Obvious tracheal mucosal edema in group B; 3) Moderate tracheal mucosal edema and marked granulomatous stenosis (increased blood vessels) in group C; 4) Cicatricial stenosis (increased Col distribution) in group D.

In order to investigate PAT for the capability of characterizing tracheal stenosis, correlation coefficients between consecutively measured PAT images on the same airway section were analyzed. The PAT measurements of were compared with histology, respectively, using correlation scatter plots. There were no significant differences between the PAT measurements and HE in groups A, B, and D, respectively, while there were significant differences between the PAT measurements and HE in group C (Supplementary Table S1).

#### MSOT Image Analysis

**FIGURE 6:**
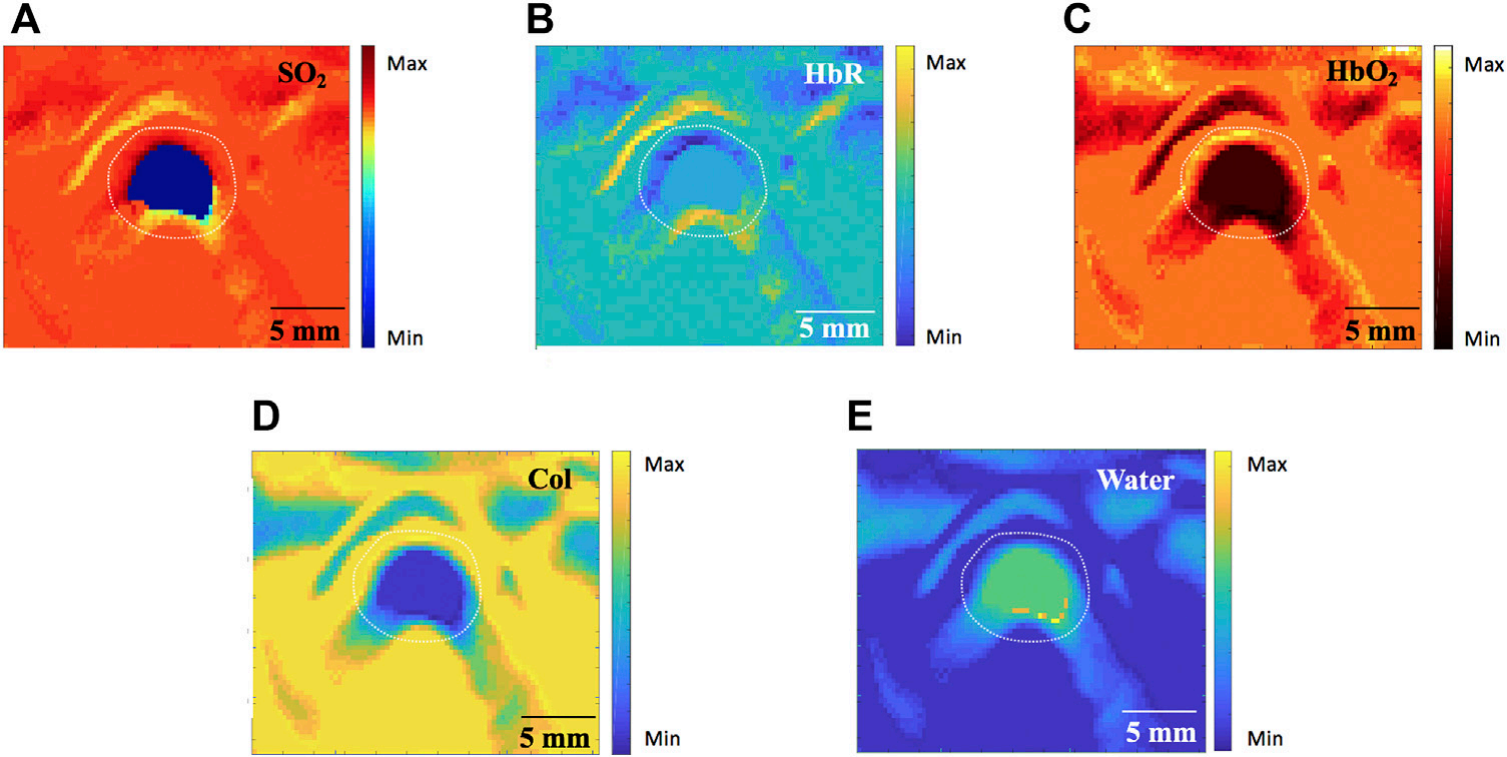
Tissue functional parameters calculated by the MSOT algorithm in Group A (760nm, 840nm and 910 nm wavelength). **(A)** blood oxygen content (SO_2_). **(B)** deoxygenated hemoglobin (HbR) **(C)** oxygenated hemoglobin (HbO_2_). **(D)** Collagen (Col). **(E)** Water. MSOT, multispectral optoacoustic tomography. Scale bars are 5 mm long.

Tissue functional parameters (SO_2_, HbR, HbO_2_, Col and water) were calculated by a multispectral algorithm. MSOT signals (Figure 6A–E) revealed SO_2_, HbR, HbO_2_, Col and water distribution in Group A, respectively.

**FIGURE 7:**
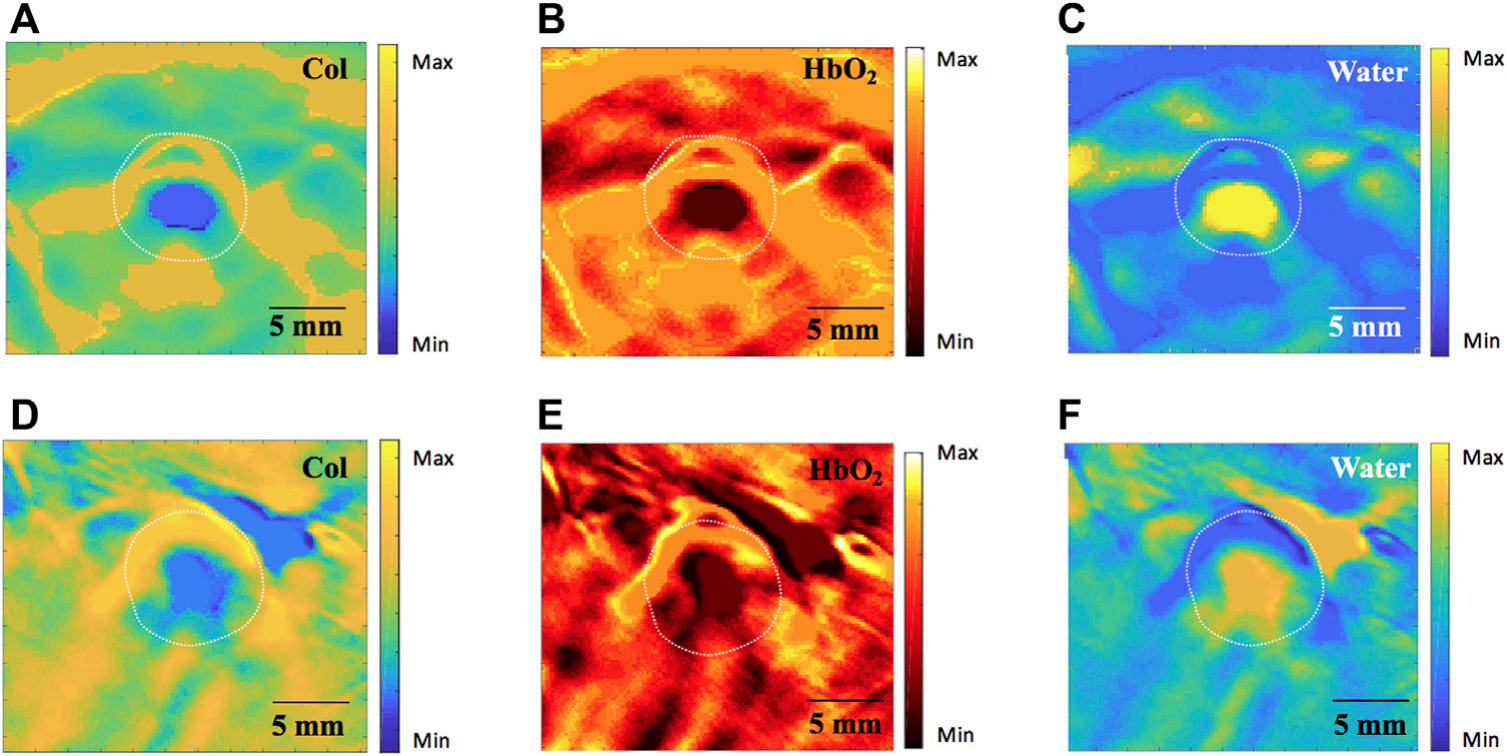
Comparison of tissue functional parameters by MSOT (760nm, 840nm and 910 nm wavelength). **(A–C)** showing Col, HbO_2_ and water distribution in group B, respectively; **(D–F)** showing increased Col, HbO_2_ and water distribution in group D, respectively. MSOT, multispectral optoacoustic tomography; Col, collagen; HbO_2_, oxygenated hemoglobin. Scale bars are 5 mm long.

In stenosis groups, MSOT signals for Col distribution in Group D were markedly higher compared to the other two groups. In contrast, MSOT signals for SO_2_, HbO_2_ and water were significantly greater in groups B and C compared to group D. Although no statistically significant differences were found between structural measurements comparing group B and D, tissue functional parameters were quite different in those two groups: 1) MSOT signals for Col were greater in group D (Figure 7D) compared to group B (Figure 7A); 2) MSOT signals for HbO_2_ (Figure 7E) and water (Figure 7F) were weaker in group D compared to group B (Figures 7B,C), respectively.

## Discussion

Accurate structural and functional analyses are crucial to assess tracheal physiological and pathological states (Ahmad and Yahaya, 2012; Both et al., 2021; Kornas et al., 2021). Rabbits represent a common animal model to study respiratory diseases in humans, consistently validated in previous anatomical studies (Ramchandani et al., 2000; Ahmad and Yahaya, 2012; Oberoi et al., 2020). Although CT (Cao et al., 2020) and magnetic resonance imaging MRI (Elders et al., 2021) are routine techniques for detection of tracheal stenosis, those methods confer a risk of ionizing radiation and/or are limited in their usage at times, as stents or metallic foreign bodies are common contraindications. Endobronchial OCT, as a new method for airway image in both animal (Mahmood et al., 2006; Lin et al., 2013; Lee et al., 2014) and human (Balakrishnan et al., 2018; Goorsenberg et al., 2018; Kozlowski et al., 2019) may cause airway mucosa injury or aggravate airway stenosis, due to its invasive detection. Furthermore, PAT and MSOT could be an accurate imaging modality for assessment of blood vessel diseases (Yang et al., 2019; Bok et al., 2021; Karlas et al., 2021), highlighting the potential of them as a new tool to evaluate structural and functional information in tubular structures like artery, vein, trachea and bowel. Here we have developed a new non-invasive PAT-based method with high temporal and spatial resolution, which is capable of acquiring structural and functional information of trachea. This PAT/MSOT system provides a high resolution optical imaging modality that exceeds the optical diffusion limit (Yang et al., 2017), and demonstrates the capability to acquire real-time images of animal tracheas.

From a structural standpoint, it was most salient to observe that PAT possesses a powerful real-time imaging ability and is able to image lumen variation in a respiratory circle synchronously, as well as the airway along the radial directions by continuous mobile scanning. Furthermore, as described above, the assessment of accuracy or inter-observer reproducibility of PAT measurements in our present study showed that there were significant correlations between PAT measurements *in vivo* and its corresponding HE in groups A, B, and D. Even if the consistency of PAT measurements and HE was poor in group C, the feasibility of our PAT method would still be valid as long as the tracheal lumen in normal rabbits (Group A) are similar to those reported study using CT (Han et al., 2009; Müllhaupt et al., 2017). This finding, however, was most likely related to the increased mucus in tracheal tissue at the fourth week, resulting in an unclear boundary between the tracheal wall and cavity, thus affecting the results of PAT. Additionally, among all the stenosis groups, the stenosis degree in group C was most severe as quantified by both PAT and HE. This may be due to the granulomatous hyperplasia in airway remodeling, which would interfere with imaging results. Nevertheless, no statistically significant difference was found between groups B and D by PAT. Both displayed progressive mucosal edema at the fourth week and scar contracture at the eighth week, resulting in moderate tracheal stenosis.

From a functional standpoint, multispectral algorithms were applied to acquire tracheal contents distribution, aiming to analyze the correlation between the MSOT images and the corresponding histology during the stenosis process. In our present study, although there were no significant differences in airway measurements between groups B and D, MSOT images indicated different tracheal contents in those two groups for increasing distribution of water and hemoglobin. This finding was suggestive of severe tracheal edema and congestion in histology. As edema is the first physiologic event that occurs with injury (Kang et al., 2015), early detection may lead to prevention and improved management of stenosis. Airway stenosis mechanisms are well recognized as reported previously (Kang et al., 2015), with the process including mucosal edema, granulomatous hyperplasia, and scar hyperplasia. Here, since lots of mucus were found in the tracheal lumen during anatomy, we speculated that the tracheal wall secreted lots of mucus in pathological state after modeling, leading to a strong signal of water content in the tracheal lumen (Figures 7C,D). On the other hand, the increasing of Col distribution in trachea were reported to contribute to the tracheal fibrotic processes in recent research (Kenyon et al., 2003; Schuliga et al., 2010; Wang et al., 2010; Junior et al., 2021; Liu et al., 2021), for which increasing subepithelial collagen deposition are important pathophysiological components in benign stenosis. Similar to our study, marked increasing of Col contents by MSOT was observed in group D, and likely attributed to the fact that increasing collagen fibers in scar hyperplasia can be observed in tracheal tissue. Notably, medications for airway stenosis are focused on how and when to block airway collagen deposition, aiming to alleviate tracheal stenosis and increase tracheal patency. As such, the potential exists for MSOT to evaluate physiological information during the tracheal stenosis process, as well as take part in therapy management.

No previous PAT or MOST studies have demonstrated its application for imaging of the trachea. In our study, not only did we image the tracheal structure, but also the functional parameters including SO_2_, HbR, HbO_2_, Col and water distribution, which the existing methods like ultrasound, CT, and MRI cannot achieve. Likewise, the entire stenosis process was imaged by PAT/MSOT, allowing accurate measurement, and thus accurate assessment for stenosis tracheal reconstruction. Therefore, our results suggest that PAT/MSOT can potentially detect trachea *in vivo* to facilitate the tracheal stenosis management.

A few limitations exist regarding this study. Given the size of our PAT probe, we could not image smaller airways in the smallest bronchioles of a rabbit model, though importantly, the size of the probe would not be as affected if our results were advanced for use in humans. In this regard, reliable bronchi PAT measurements could not be achieved in our present study. Additionally, although image quality was considered satisfactory, it could be improved by a wider range of wavelengths, such as 1,000–1,200 nm, which may provide more physiological and pathological information of tracheal tissue.

## Conclusion

This study was designed to demonstrate the feasibility of PAT/MSOT in detecting disease-induced tracheal stenosis. Given the real-time and high spatial resolution capabilities, PAT/MSOT has the potential to become a useful tool for non-invasive airway imaging in future. We believe this study sets the ground work for the analysis of the trachea using PAT/MSOT, especially given its ability to capture important information regarding disease pathophysiology. We highlight that further studies need to be performed to demonstrate the ability of PAT/MSOT to assess more sophisticated information in the pathologic condition of tracheal stenosis, including tracheal wall mechanical properties and tracheal patency. We hope to pursue such studies in the future and develop a PAT-based modality to better non-invasively detect, diagnose, and monitor the trachea in humans.

## Data Availability

The original contributions presented in the study are included in the article/Supplementary Material, further inquiries can be directed to the corresponding author.

## Supplementary Material

Supplementary Table S1 Comparison of PAT Measurements and the corresponding histology.
