# Supplementary material for: Evaluation of Tracheal Stenosis in Rabbits Using Multispectral Optoacoustic Tomography
Source: Front Bioeng Biotechnol. 2022 Mar 4;10:860305. doi: 10.3389/fbioe.2022.860305 (PMC8931196; doi:10.3389/fbioe.2022.860305)
Supplement: Supplementary file 1 [file Table1.DOCX]

**Table 1. Comparison of PAT Measurements and the corresponding histology**

|  | **TWT (mm)**  **Obs 1 Obs 2 HE** | **CSA (mm^2^)**  **Obs 1 Obs 2 HE** | **SR (%)**  **Obs 1 Obs 2 HE** |
| --- | --- | --- | --- |
| **Group A** | 0.82±0.01 0.82±0.04 0.77±0.07 | 16.28±0.36 16.52±0.30 17.46±0.82 | — — — |
| **Group B** | 1.14±0.04 1.14±0.05 1.07±0.47 | 12.28±0.31 12.61±0.26 13.59±0.40 | 61.43±0.98 60.43±0.82 57.33±1.23 |
| **Group C** | 1.50±0.52 1.53±0.05 1.29±0.26* | 8.74±0.73 8.80±0.48 10.99±0.73* | 72.55±2.31 72.38±1.51 65.49±2.29* |
| **Group D** | 1.17±0.03 1.17±0.20 1.09±0.08 | 12.44±0.25 12.53±0.19 13.73±0.70 | 60.92±0.79 60.69±0.60 56.91±2.20 |

TWT: tracheal wall thickness, CSA: cross-section area, SR: stenosis rate, PAT: photoacoustic tomography, Obs: observer, HE: histologic examination. *p<0.05
